# A Compact IIoT System for Remote Monitoring and Control of a Micro Hydropower Plant

**DOI:** 10.3390/s23041784

**Published:** 2023-02-05

**Authors:** Anca Albița, Dan Selișteanu

**Affiliations:** 1 VIG IMPEX Ltd., 200129 Craiova, Romania; 2 Department of Automatic Control and Electronics, University of Craiova, 200585 Craiova, Romania

**Keywords:** monitoring, wireless communication, remote control, electric power plant, software application, IIoT

## Abstract

Remote monitoring and operation evaluation applications for industrial environments are modern and easy means of exploiting the provided resources of specific systems. Targeted micro hydropower plant functionalities (such as tracking and adjusting the values of functional parameters, real-time fault and cause signalizing, condition monitoring assurance, and assessments of the need for maintenance activities) require the design of reliable and efficient devices or systems. The present work describes the design and implementation procedure of an Industrial Internet of Things (IIoT) system configured for a basic micro hydropower plant architecture and assuring simple means of customization for plant differences in structure and operation. The designed system features a set of commonly used functions specific to micro hydropower exploitation, providing maximum performance and efficiency.

## 1. Introduction

The use of data acquisition systems for monitoring and control of specific events is currently widespread in most industries, even for estimation of the degree of wear and the development of maintenance programs for specific devices and systems. Specific software applications for data acquisition systems allow for fast identification of untimely faults, as well as their repair. The basic principles of data acquisition implementation have been applied in various architectures and successfully adapted to the various particularities of the implementation environment [1]. Medicine [2], automotive [3,4], and electric power industries [5,6] are just a few fields of activity in which the features provided by data acquisition systems are essential.

The acquired data are gathered for further analysis by an intelligent processing unit such as a data concentrator or a server, in most cases, serially collecting the required information. Although a handy and stable means of local data transfer, an increasing number of situations require remote monitoring, control, and data acquisition, requiring additional communication support. Optical fiber has been proven a suitable a solution for systems implementing applications designed to meet certain industrial control standards [7].

In modern remote communication design, the use of IoT systems is spreading due to their ease of operation, provided features, and versatility. Low-cost IoT alternatives are currently implemented in domains such as medicine [8], industrial monitoring and control applications [9,10,11], and smart home automation [12]. Due to permanent demand and development of such remote solutions in industrial environments, especially in the energy field, a distinct set of characteristics and implementation principles define applications in the sphere of the Industrial Internet of Things (IIoT) [13]. The increased control and freedom in implementation provided by IIoT technology and specific applications lead to their inclusion in the development of systems, sensors, and devices with specific functions and improved efficiency. For example, IIoT sensors and open-source solutions can be used to measure gas waste in brownfield production assets from a manufacturing facility as a step forward in finding means to reduce operating costs [14].

Various approaches in IoT and IIoT implementations can be chosen for applications depending on the targeted performance and requirements. Narrow-band IoT monitoring systems can be used to implement an efficient microgrid power quality monitoring solution [15]. Low-cost IoT systems can also be designed for conditional monitoring of induction motors [16]. In a more complex approach, smart maintenance can be performed through conditional monitoring of rotary equipment via an automated data collection, processing, and interpretation system based on an IIoT framework [17]. Moreover, IIoT technologies are often combined with other approaches so that the designed system provides the most suitable solution for the problem at hand. The authors of [18] proposed cloud technology that complements IIoT modem features in a wastewater monitoring system signalizing unexpected wastewater inlets. Machine learning techniques were used to help IoT technology in the implementation of a supervisory, control, and data acquisition system for automatic fault detection, improving predictive and preventive maintenance and reducing breakdown times [19]. 

The design of many problem-specific IoT applications implementing various and handy protocols can lead to an impasse regarding the communication in wireless sensor networks due to the lack of standard-based network activity. A wireless sensor network architecture was designed in [20] with the aim of eliminating such limitations.

IIoT solutions have recently gained popularity in the electric power domain by adapting these technology features to various targeted applications. Hydropower facilities specific to micro power plants have an architecture assuring the most efficient use of water as a primary resource. In this regard, a water tank is set upstream to collect water from the surrounding area. Meanwhile, several micro hydropower plants are installed downstream (approximately 10 km away, depending on geographic situation). The control of both types of resources is managed by a single control point placed either within the facility or at the headquarters of the managing company. This organizing mode requires the use of remote-control applications, most often implemented on GSM support. Such applications are suitable for the management of either a single micro hydropower plant or for a whole hydropower facility.

The proper functioning of a hydropower facility disposing of micro power plants and lacking remote-control features implies either the existence of an operator for each component micro hydropower plant or a single operator periodically inspecting all the micro power plants. Both of these approaches lead to cost disadvantages (operator payment and inspection trip costs) and micro hydropower chain malfunctioning, as fault events cannot be rapidly removed (leading to machinery blockage or water discharges). As a consequence, real-time monitoring of parameters for each micro hydropower plant, signaling of faults, and their rapid removal allow for the optimal functioning for each micro plant, as well as of the whole hydropower facility. A solution providing these features represents an advantageous way to modernize micro hydropower plants in conventional construction, enabling the adaptation to current requirements with minimum costs. Meanwhile, the life span of electromechanical components is preserved, as they become unusable should the plant be modernized through integrated alternatives.

Configuring a supervisory, control, and data acquisition (SCADA) system for a micro hydropower plant requires an exceedingly complex solution, as many of SCADA features are redundant in this case. Moreover, the implementation cost for such systems, including, perhaps, an IIoT implementation able to solve the communication issues analyzed in [21] is not justified in this context. A compact and adaptable approach is therefore preferred. Our experience in implementing data acquisition systems for industrial environments, especially in electric power system applications [22,23] and IIoT-aided remote monitoring [24], justifies the design of an IIoT system providing the best performance under the described conditions.

Therefore, it can be established that a system assuring the basic monitoring and control requirements for a conventional micro hydropower plant must satisfy several conditions:

- Real-time monitoring of critical controlled plant parameters;
- Wireless communication for monitored plants;
- Storage of local value archives for monitored parameters and plant event logs for start-ups, shutdowns, and faults;
- Performance of basic commands (stopping or starting the micro hydropower plant generator);
- Provision of feedback when performing computer-launched or plant-generated commands.

The system presented in this paper provides a modern and versatile solution for remote monitoring and control, assuring wireless communication through GSM support and implementing a message dialogue based on the Message Queuing Telemetry Transport (MQTT) protocol. As a clarification for the readers, this system illustrates a different approach to using common IIoT tools (such as artificial intelligence), instead focusing on embedding customized IIoT features to assure stable and performant remote communication. As data transmission is a critical issue in industrial data acquisition systems, although no special complexity of data processing is achieved in this case, IIoT techniques and specific hardware can be included in an industrial data acquisition system architecture. To justify this statement, in the present work, we focus, in particular, on describing a properly implemented hardware structure and ensuring safe communication. 

The variety of information characterizing a micro hydropower operation mode and the complexity of acquiring and managing its specific parameters suggest the use of a data concentrator that is versatile and reliable enough to allow for both direct data acquisition for a diversity of signal types and communication with other devices from the micro hydropower plant structure. The same architecture must enable commands received from the superior hierarchical level of control to be performed, mainly through digital outputs. The data concentrator, together with the high-level hardware structure and their implemented software application, must correctly perform real-time monitoring and control of the managed structure within the established allowed time limits for the tasks. Equipping the system with hardware to enable the implementation of IIoT communication technologies in a customized application suite can be considered an adequate solution. Compensating the timing limitations by conditioning the data transfer, in addition to providing user feedback while performing commands, would maintain the system stability and its correctness in operation. If such an approach satisfies the performance requirements, no additional advanced solutions or configurations for data processing are needed. Hence, improved function and configuration independence are assured by solely using a data concentrator, a software application for control, and a hardware and software core designed for wireless communication.

Unlike an integrated solution, the system can be configured to monitor and control various micro hydropower architectures. The designed application implements a remote monitoring and control core for a micro hydropower plant and can be easily adapted to the particularities of all the plants from a micro hydropower facility, as any plant without an integrated wireless communication infrastructure is eligible for this approach. The hardware design and components of the system are presented in Section 2. In Section 3, we describe the system’s software structure and implementation process in detail. Several implementation issues, as well as methods for their resolution compared to other approaches, and some extended improvements are highlighted in Section 4. In Section 5, we present the system operating mode and report results obtained while performing functioning tests at an existing micro hydropower plant that is part of a real hydropower facility. Finally, conclusions and various development directions with respect to solution versatility are reviewed in Section 6.

## 2. Hardware Structure

As the main requirements for the designed system have been established, it can be concluded that the hardware and software solution to be implemented must provide means for monitoring the hydrogenerator currents and voltages for the implementation of a basic set of protections. Moreover, the system must provide a means of acquiring the data of interest from other possible specific structures included in the plant, such as temperature or rotational speed sensors. The essential remote-control function must be assured through wireless communication infrastructure involving microsystems providing GSM modems. Several functional units have been established to manage all the necessary hardware architecture. The system can be organized around two sections, i.e., a control section and an executing section located in the two areas corresponding to the situation encountered the field. The first is located at an intermediary command point, and the second is located within the micro hydropower plant architecture.

Regarding the executing component of the system, an efficient infrastructure consisting of industrial devices for data acquisition and control can be configured by using a specialized unit to monito hydrogenerator currents and voltages, in addition to performing complex protection functions. These features can be successfully performed by the *PC-05/104 Process Control* [25] industrial unit. The provided data are then transferred to a data concentrator capable of acquiring all the other functional parameters of the micro hydropower plant either directly or by connecting to specific measurement blocks. The *PC-06/104 Process Control* industrial system [26] is adequate for this functionality, also providing serial transfer to the higher hierarchical level for globally stored data.

As the most versatile and efficient remote communication means is wireless, GSM support is implemented using performant modems suitable for industrial environments. IFB-122 units [27] are intelligent structures equipped with a GSM modem [28] and running a Linux operating system to ensure those features.

The above-described collection of units and devices established for the implementation of the monitoring and control system under discussion is summarized in Table 1. 

**Figure 1:**
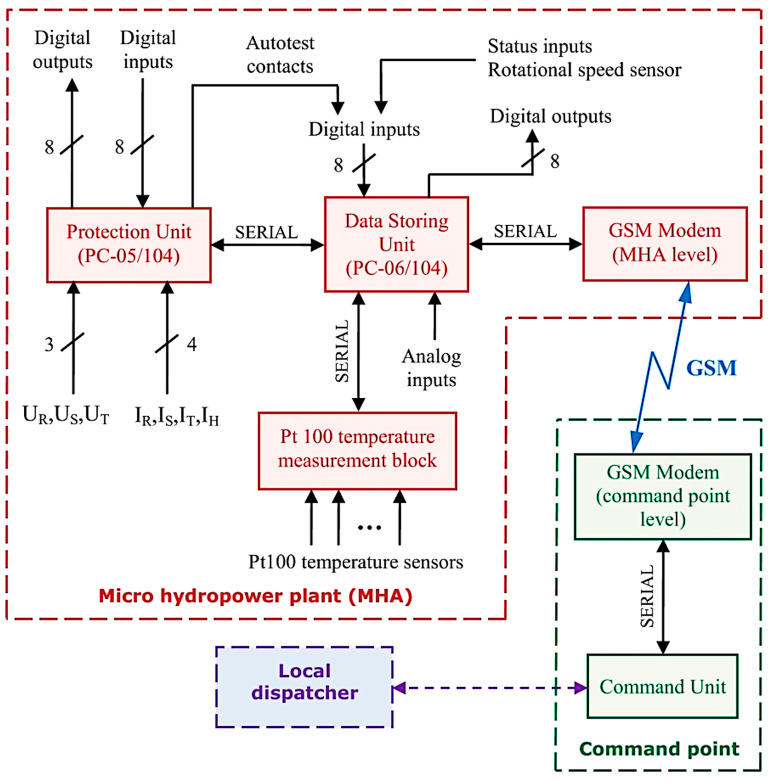
Hardware structure for the proposed remote monitoring and control system.

The hardware structure was designed based on a practical architecture that already exists in the field. However, it can be used with minimal adaptations to any other conventional micro hydropower plant configuration with similar resources. A diagram of the implemented architecture including the described units is presented in Figure 1. The role of PC-06/104 as a data concentrator (data storing unit) can be easily noticed, as all electrical and non-electrical quantities characterizing the function of the micro hydropower plant are acquired at this level. Some of these quantities are collected serially through PC-05/104 (protection unit) and PT 100 (temperature measurement block), whereas others are directly connected to PC-06/104 as analog inputs and directly acquired from various transducers. The GSM modems are located at the micro hydropower plant (MHA) level and at the command point level. 

Serial reading commands from the command unit are sent to the modem and transferred through GSM support to the MHA level, where the correspondent modem receives the information and transfers it serially to the data concentrator to further receive the response the concentrator sends back to the command unit. When a command is initiated, the request is sent in the same manner, with data concentrator performing the command by triggering its corresponding digital output.

**Figure 2:**
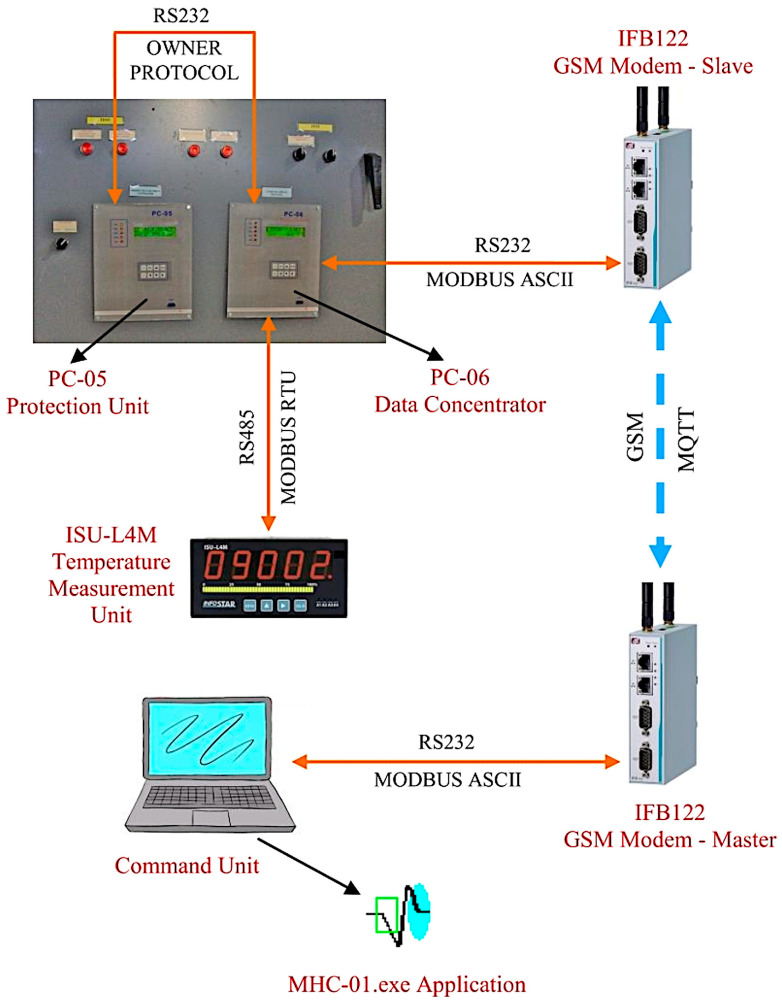
System communication flow.

Designing and implementing a hardware infrastructure allowing for remote management of a micro hydropower plant functioning from a command point situated 8–10 km away represents the fundamental feature offered by this system. Apart from a well-structured architecture for acquisition of the necessary data and a compact and easy means of control, an overall effective supervision of plant operation is thus provided. The existence of a robust and efficient communication infrastructure able to manage the various forms of information exchanged between the system nodes is of critical importance for the proper functioning of the implemented structure. An overview of these interactions is presented in Figure 2.

As shown in Figure 2, the command unit sends requests through the RS-232 serial support and the MODBUS ASCII data protocol to the afferent GSM modem (master). The Master modem acquires the serial data and transfers them through the MQTT protocol to the correspondent GSM modem (slave) at the micro hydropower plant level. The slave modem serially retransmits the MQTT message as a command to the data concentrator (PC-06). The protection unit (PC-05) provides information to the data concentrator through the serial RS-232 owner data protocol, and the temperature measurement adapter (ISU-L4M device [29]) provides data through the serial RS-485 MODBUS-RTU standard protocol. Meanwhile, the data concentrator decodes both types of information, in addition to gathering and processing the rest of the data, and transfers the global information through serial RS-232 support and the MODBUS ASCII standard data protocol. Although hardware compatibility is assured through the design of the different communication supports, proper data transfer is managed by the system’s software suite.

## 3. Software Applications

The implemented hardware structure must be easily and efficiently managed by a specific software suite, including applications designed to satisfy all aspects of interest regarding the control and basic supervision of a micro hydropower plant. Therefore, the software suite designed and implemented for this system contains:

- A high-level software application (*MHC-01.exe*) with the role of displaying the centralized acquired information, real-time monitoring of specific element behavior, and assuring their control, which was developed in the Visual C++ environment and runs on Windows operating systems;
- Firmware consisting of two complementary applications (*mhc-Master.exe* and *mhc-Slave.exe*) developed at the GSM modem level to manage wireless communication implemented using C++ programming language and running on a Linux operating system.

The PC-05/104 and PC-06/104 units also operate with specific software applications designed and implemented to assure serial data transfer according to the established protocols depending on the commands received from the command unit, required protocol conversions, and temporary data storage. PC-06/104 firmware also allows for optional configurations, providing a customizable register structure for storage of the acquired data.

### 3.1. MHC-01 High-Level Software Application

The software application (*MHC-01.exe*) running at the command point location features interactive means of displaying and refreshing the real-time monitored signals and events occurring at the micro hydropower plant level. The software provides a collection of schematic diagrams specific to the structure of the monitored plant. A basic set of commands and signalizations is also provided. Moreover, the application features an event log that is updated with each triggered command, in addition to providing essential parameter values that are acquired when the event occurs. Lastly, an extensive archive listing most monitored parameter values is created as MHC-01.exe is operating.

This collection of functionalities is carefully implemented and managed within a Visual C++ application, which organizes them in three important modules:

- The **graphical interface module** builds the panel set illustrating the connected schematic diagrams defining the plant structure; specific software objects are also established within this module to manage data updates and the graphical dynamic of the windows corresponding to ongoing events;
- The **communication module** handles the timed data update through a generated thread, which includes the request/response dialogue between the command unit and the PC-06/104 unit;
- The **data refresh unit** organizes the information required for graphical interface refreshing using a data structure containing the values stored and transferred by the PC-06/104 unit and acquired from the registers of the interrogated data acquisition units into its own register structure.

**Figure 3:**
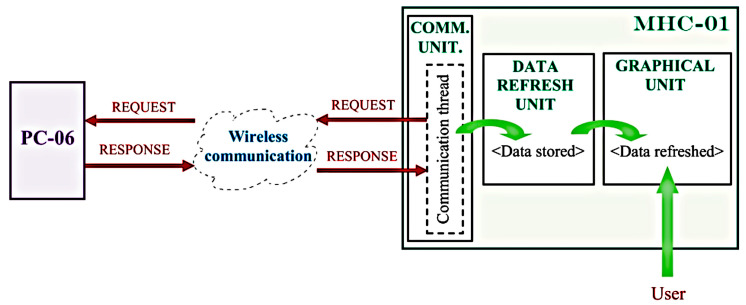
MHC-01 module interaction.

Figure 3 shows the module interaction within the MHC-01 project through a suggestive diagram.

As shown in Figure 3, parameter values are periodically refreshed and displayed according to the schematic diagram to which each is related. Each command unit timed request is sent using the wireless infrastructure to the PC-06/104 unit (PC-06), the response of which triggers data update and storage (through *Communication Thread* and *DATA REFRESH UNIT*) and the timed refresh of the *GRAPHICAL UNIT* according to the panel selected by the *user*.

### 3.2. GSM Modem Firmware and Wireless Communication

The wireless communication infrastructure for the presented system is assured by IFB-122 units for data processing and control equipped with GSM modems. The two systems are situated at the micro hydropower plant level (MHA) and at the command point level (command unit), respectively. Their implemented firmware enables dialogue between the PC-06/104 firmware and the MHC-01.exe application. It is imperative for the same firmware to implement the wireless communication characteristics of an IIoT system, namely:

- To implement an IIoT-specific communication protocol; after a thorough analysis for all the possible communication scenarios and the structure of the transferred information, it was concluded that a message queuing telemetry transport (MQTT) protocol, together with its provided library (*libmosquitto.dev* for a Linux operating system), can satisfy the imposed requirements;
- To allow an Internet broker interaction to direct messages between the two terminals;
- To implement specific publish/subscribe functions to assure dialogue between the monitoring- and-control-responsible element and the controlled element;
- To provide the necessary communication and data transfer security features;
- To assure fast, safe, and robust data communication.

**Figure 4:**
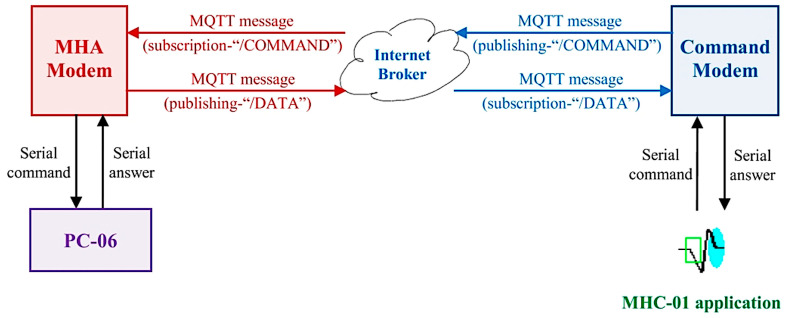
Wireless communication and modem interaction.

Figure 4 illustrates the solution to meet these requirements into a concise diagram. According to this diagram, the serial dialogue between the two terminals (*PC-06* and the *command unit*) is extended through the wireless hardware and software infrastructure assured by the *command modem* and *MHA modem*.

According to this basic scenario, the two complementary applications representing the firmware must be developed and tested in parallel, each dealing with distinct issues of the implemented master/slave dialogue. It can therefore be observed that at the master level (command unit), as the serial command initiator, the publishing of the command message is conditioned by whether serial data have been read or not. Meanwhile, the publishing of the response message from the slave level (PC-06) is conditioned by the of at least one subscription message. Furthermore, the subscription approach differs between master and slave. 

**Figure 5:**
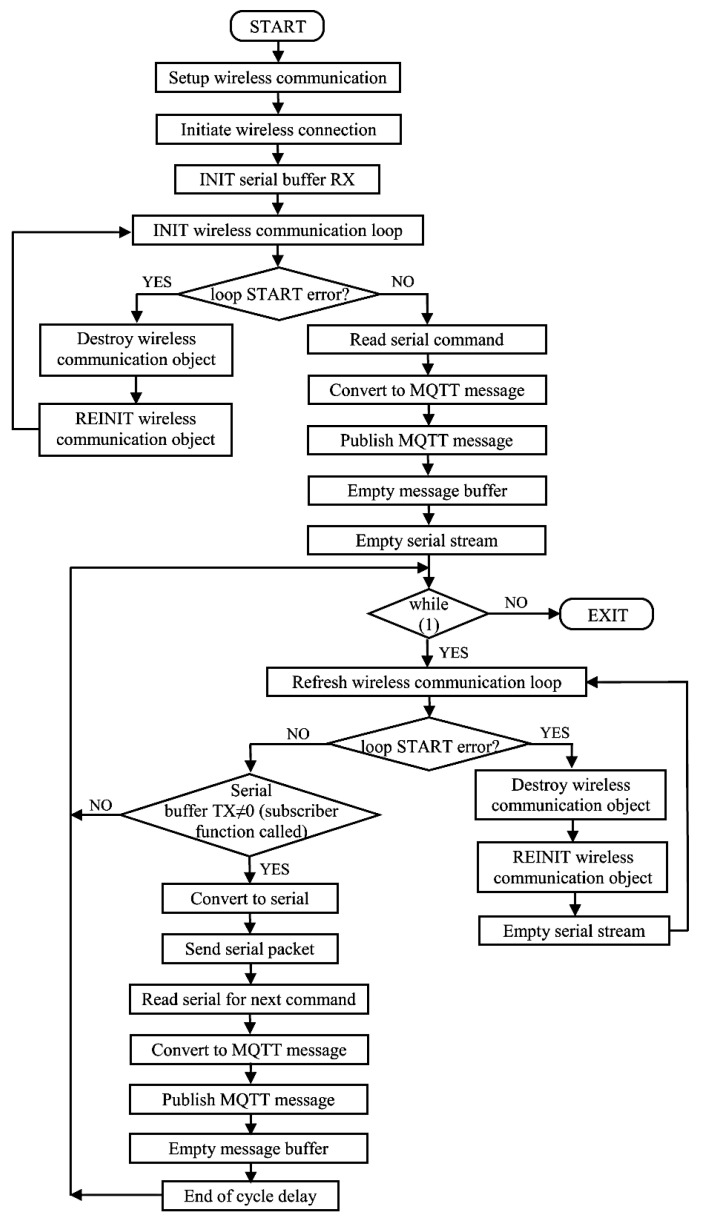
Main loop flow chart for master firmware.

The master modem firmware treats the request/response sequence as follows. The subscriber function (*OnMessage()*) converts the ASCII message into a binary data buffer. The serial transfer of this buffer is performed in the corresponding *main()* function, which is conditioned by the existence of a valid binary data buffer, thus assuring that the response from the previously published command has been received from the command unit (through the *OnMessage()* function call, which leads to the completion of the binary data buffer). The specific loop implemented within the master application is shown as a flow chart in Figure 5.

**Figure 6:**
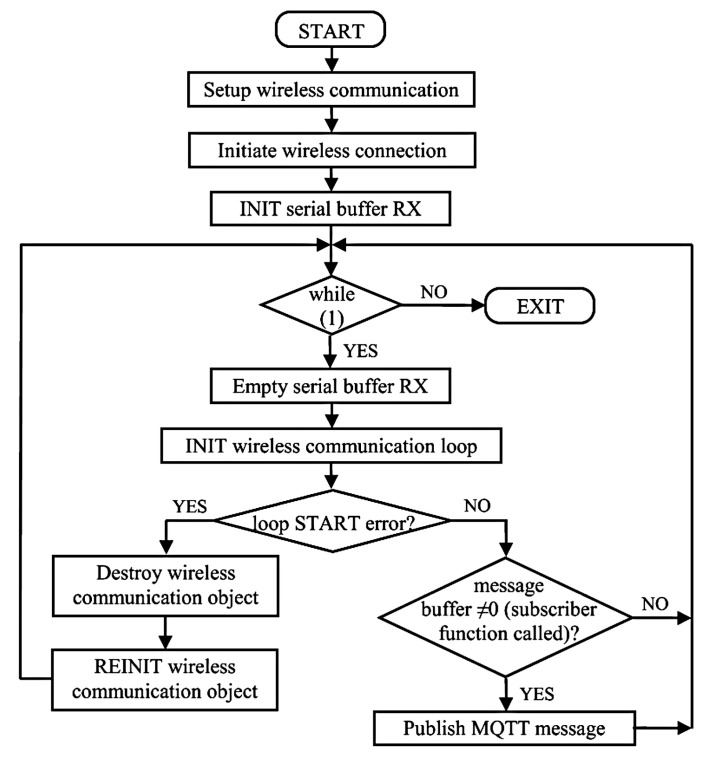
Main loop flow chart for slave firmware.

Meanwhile, the slave (MHA) modem firmware approaches the request/response sequence differently. The application converts the subscribed message into a binary data packet within the correspondent *OnMessage()* function, then sends the binary request to the PC-06 unit and receives the response, converting it into an ASCII message and saving it in a buffer, pending its publishing. In this case, the message is published within the *main()* function if a completed message buffer exists. This condition establishes that the call of the subscriber function always occurs before publishing a new message. The corresponding implementation functionality is illustrated as a flow chart in Figure 6.

**Figure 7:**
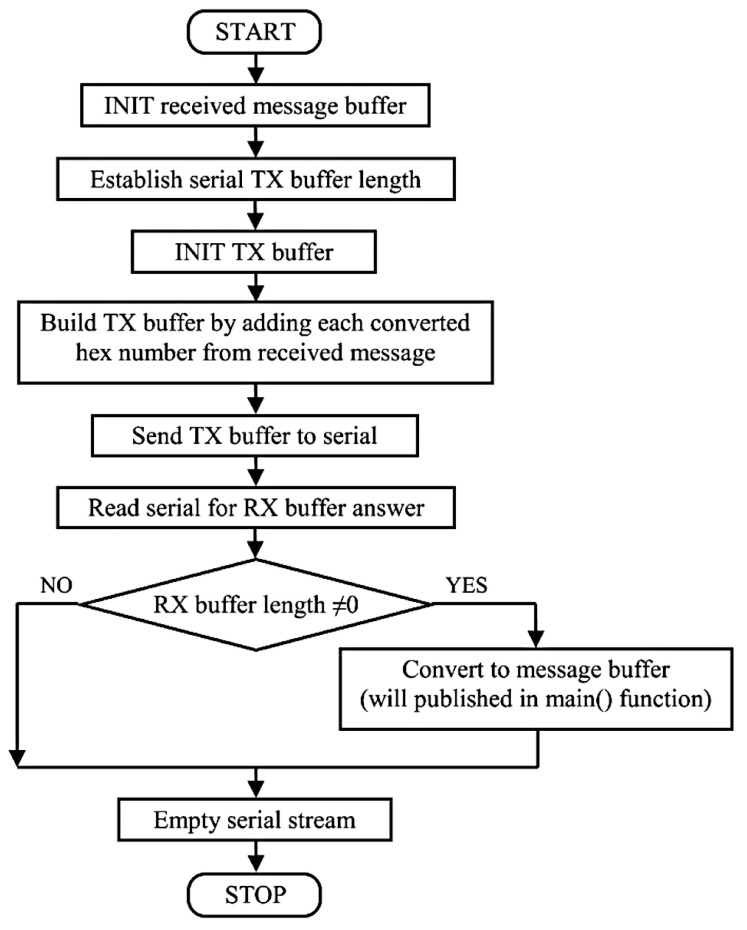
Slave *OnMessage()* subscriber function flow chart.

Furthermore, a flow chart illustrating the specific structure of the slave subscriber function is presented in Figure 7. According to the diagram, the *OnMessage()* function successively performs a set of actions, including the building of the request packet (TX buffer), reading of the response packet (RX buffer), and saving the RX data within a message buffer.

## 4. Implementation Issues 

The current trend in IoT application development implies a hardware configuration consisting of a collection of IoT sensor nodes and a managing software for their customization, control, and individual software development support, as presented in [30]. The development of an IIoT data concentrator to transfer the measurements from simple devices to free web technologies for analysis is another approach explored in [31]. Complex studies of extended solutions for real-time anomaly detection using IIoT technology, cloud computing, and edge AI, such as those presented in [32], have been proposed using the same model of application, in which IIoT servers collect data from smart devices, which are exchanged through secured protocols through edge and cloud databases. The compact solution for micro hydropower plant management allows a different design in which the data concentrator provides various means of serially or directly gathering the data, assuring overall information coding according to the establish data protocol. Meanwhile, the software application for monitoring and control performs offline correspondent information decoding, as the firmware on the GSM modem level focuses on the transfer of coded data. In this manner, the wireless system has a maximum level of versatility, running independently of the implemented data protocol. Moreover, industrial infrastructure without IoT devices can be much more easily connected to a conventional serial data concentrator. In this manner, a large portion of the hardware limitations encountered in a more complex implementation manner is eliminated. 

Furthermore, performance requirements such as fast command response, timed and fast data refresh, and a stable wireless connection, from both a hardware and software point of view, need to be ensured. At the hardware level, given that at least one of the GSM modems is located at the plant level that is specially adapted to industrial environment systems or devices must be used. IFB-122 modems are designed to operate in extended temperature ranges and ensure resistance to disturbances. Moreover, although the infrastructure is tolerant to medium-quality GSM signals, not requiring intense data traffic, the utilized GSM modems enable the connection of two GSM antennae in case of deterioration of the communication conditions. 

From a software point of view, the firmware implemented in the IFB-122 structures must assure, for both master and slave applications, safety reconnecting possibilities in the case of communication errors, such as:

- The modem is disconnected from the broker with which it exchanges messages;
- The GSM signal is lost (the modem is disconnected from the wireless network);
- Hazardous events occur, such as stopping the command unit application from the master terminal, or the PC-06 firmware freezes or stops; this case generates a waiting state for both the master and slave (the master awaits a new serial command, and the slave waits a subscription message), with eventual reconnections due to the lack of activity in the wireless network;
- A wireless communication environment initiation error occurs in sending messages; in this situation, destroying and recreating the MQTT communication object, along with its reconnection to the broker, represent the most handy and safe method to repair the wireless communication context.

**Figure 8:**
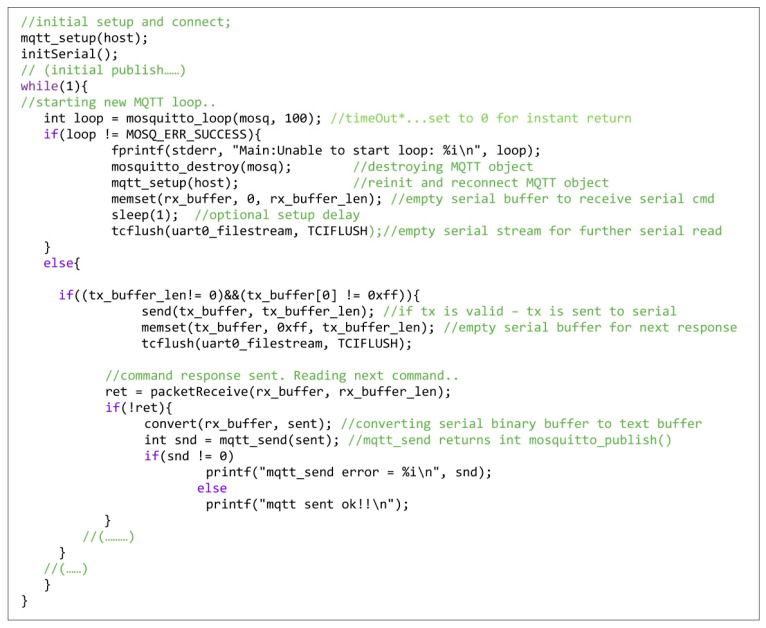
Dealing with *mosquitto_loop error* in the master firmware main loop.

A solution to dealing with this kind of error from the software implementation point of view is listed in Figure 8 through a code sequence extracted from the firmware application of the master modem. The reinitialization process caused by the loop error (*mosquitto_loop error*) includes several operations: saving the notification error, destroying the MQTT object, resuming the setup process, and preparing the reception buffer to store the serial command. On the contrary, if the loop is successfully refreshed, the program waits for the transmission buffer to be completed, in which case a new command is read and published after the serial transfer of the previous response. In the complementary slave modem application, this type of error is managed in a similar way.

Adapting the MQTT message communication that is suitable for broadcast to a request/response-based serial communication, which the first must emulate, implies a specific setting configuration and a predefined order for message publishing, with adequate conditioning in the correspondent subscriber function. From the message flow point of view, a one-on-one correspondence between the request message and the response message is mandatory. This is the only way of assuring the expected behavior of the PC-06 unit with respect to the requests sent through the MHC-01 application. In this context, the request messages that did not receive a timely response are set to be ignored, leading to a serial TIMEOUT behavior in the command unit for the sent request. Hence, *QOS1* is preferred for the published messages, as it has been proven suitable under the imposed communication conditions. The broker message queue is also ignored, as the subscriber function only processes the currently received message. 

Message synchronization is also assured by conditioning the call of the subscriber function upon the appearance of a single published message. In this manner:

- At the master level, the publishing of each request message is triggered by the reception of the previous response;
- At the slave level, the publishing of each response message is triggered by a previously received message from the master.

Regarding communication safety, mainly implying wireless communication, there is a diversity of data security methods that can generally be implemented for IIoT applications. Apart from the usual security approaches implied by the MQTT message transfer protocol, an efficient three-phase authentication protocol for IIoT wireless sensor networks including a PUF chip to ensure the physical security of the devices can be applied for critical data security in IIoT systems, as presented in [33]. 

With respect to the compact system considered in this work, it can be observed that the transferred data that circulate in the implemented application are binary encrypted according to the implemented data protocol. The significance of the decrypted information is specific to the monitored structure. Thus, the corresponding encryption and decryption is performed offline at the terminal level (command unit for initiative and data concentrator (PC-06) for execution and response, both of which are represented in Figure 1).

**Figure 9:**
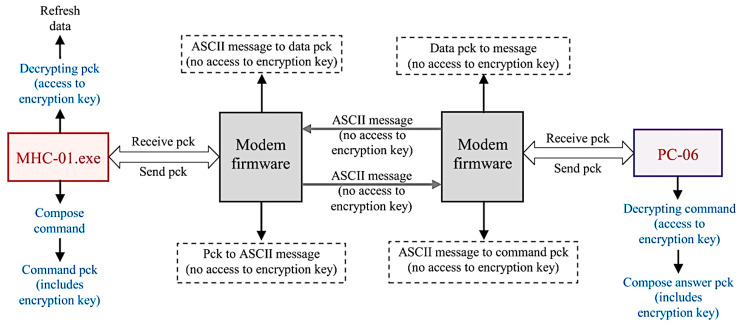
Data accessibility between the control (MHC-01.exe) and execution (PC-06 system) units.

Separating the encryption/decryption process from the wireless data transfer process and using the Linux-compatible firmware suite available on the GSM modems as a software emulator for serial communication provides several advantages. First, the Internet-transferred data have already undergone a sufficiently rigorous encoding process (binary encryption followed by the ASCII conversion requested by the MQTT protocol), the key of which is inaccessible at this level (MQTT-transmitted data are irrelevant without the corresponding context, as the values and what they represent refer to monitored structure-specific information, the configuration of which can only be found offline at the terminal level and not at the GSM modems connected online). Moreover, this encoding process allows for supplementary encryption to increase cybersecurity in other situations if necessary. Secondly, the two firmware applications minimally implemented on a Linux operating system concentrate on assuring communication stability, customizing the software instruments provided by *libmosquitto* for the analyzed practical solution, and creating specific algorithms to successfully manage the two-terminal dialogue. In this manner, MQTT involvement in decoding the data remotely transmitted through the Internet is avoided, as well as protocol vulnerabilities. In summary, the discussed levels of encryption, decryption, and decoded information availability are presented in Figure 9.

This hardware and software organizing strategy has already been tested in several applications implementing bot owner protocol communication and standardized communication.

Therefore, it can be concluded that managing the decoding process and information gathering apart from the implemented wireless communication mechanism already assures sufficient data privacy for a set of information specific to the signals of the monitored plant. Furthermore, using different topics for master (/COMMAND) and slave (/DATA), along with the use of an owner broker, provides—in addition to privacy—a better organized message interchange by classifying and ordering their delivery at the broker level. Stricter data control can be further developed easily within the master and slave modem firmware either by implementing a dynamic topic mechanism or by further codifying the sent or received message. Authentication methods and the use of private and public keys are also a means of increasing data security if necessary.

## 5. Operating Mode and Functional Testing Results from an Existing Micro Hydropower Plant

According to the establish requirements, it is of critical importance that the designed hardware structure and the corresponding software applications correctly implement a collection of real-time tasks fitting one of the following categories:

- *Periodic tasks*, which are represented by the refresh sequences for various information types provided to the graphical interface and registered in the archive files. A collection of such tasks performs a full refresh of the data gathered by the implemented system. Such updates occur often enough to record all state changes and parameter value modifications but rarely often enough to allow for both the data acquisition and wireless transfer. The testing conditions for the implemented system consider a complete update sequence performed approximately every 20 s and an archive entry registered every 1 min as proper time intervals;
- *Spontaneous tasks*, which occur upon user initiative, such as the events generated by the generator start-up/shutdown commands or increasing/decreasing the delivered power. To supervise their execution, confirmation messages are implemented at the graphical interface level. These messages, which warn for a delay of 30 s before the command is performed and the command panel is refreshed on the graphical interface, prevent the user from simultaneously launching commands.

The task periodicity and command performance duration are tolerant time delays established at the high-level command unit. In this manner, the real-time behavior can be managed by the high-level software application running on a Windows operating system based on the way this aspect is dealt with in a monitoring structure using serial communication. This solution allows for a sufficiently rigorous control, in addition to fitting the time limits set for the implemented system. 

**Figure 10:**
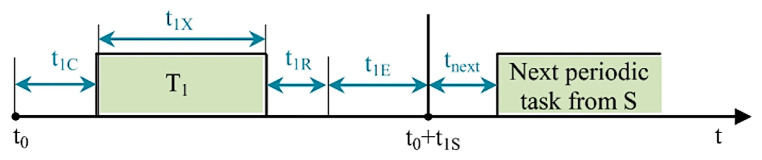
Timeline for the periodic data refresh task.

For example, let us consider the periodic task (T_1_) from the update sequence (S). Whereas the serial transfer time is considered insignificant compared to the wireless data transfer, the moment when T_1_ is launched is marked by t_0_, the wireless data transfer (to the data concentrator terminal) delay is represented by t_1C_, the terminal execution time for T_1_ is denoted as t_1X_, the wireless transfer time of the response for the command unit is marked by t_1R_, and the remaining time until the next task from sequence S is indicated by t_1E_. The wireless command transfer time for the next task in sequence S is marked as t_next_. The associated diagram is presented in Figure 10.

Given the notations, the execution time for T_1_ launched at moment t_0_ is expressed as:

t_1S_ = t_1C_ + t_1X_ + t_1R_ + t_1E._ (1)

If t_1R_ expires, the additional t_1E_, which includes the timeout iterator, is further granted for the response. If the number of iterations reaches the established timeout, the interface update contained in T_1_ is skipped, and the next task from sequence S is initiated. If all tasks from sequence S reach timeout, sequence S is restarted. Two timeout situations for all the tasks from sequence S lead to activation of a flag stating that the communication between the two terminals is considered interrupted. Reestablishing the communication implies launching a new data refresh command. It is important to mention that in this case, a task (t_k+1_) following t_k_, both of which are included in S, is programmed to wait until the correspondent t_kE_ expires before being launched.

**Figure 11:**
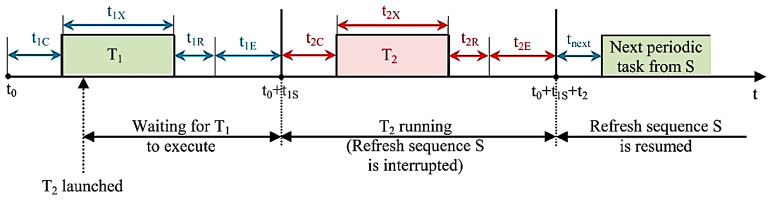
Timeline of a spontaneous task occurring during the update sequence.

Meanwhile, if spontaneous task T_2_ is launched while sequence S is periodically running, the execution time of T_2_ depends on the moment from T_1_ execution when this task is initiated. From the established priority point of view, it is noted that T_2_ occurs prior to sequence S but not prior to T_1_; hence, the execution of T_2_ does not occur until T_1_ has finished its execution. Figure 11 shows the described task execution scenario, noting that t_2C_ is the corresponding wireless transfer command to the data concentrator time, t_2X_ is the execution time for T_2_, t_2R_ is the wireless transfer response to the command unit time, and t_2E_ is the remaining time associated with the command performed at T_2_. 

According to these notations and assuming that t_1X′_ is the moment at which T_2_ has been initiated, the T_2_ execution time (t_2_) can be calculated as:

t_2_ = t_1X′_ + t_2C_ + t_2X_ + t_2R_ + t_2E_ (2)

As Figure 11 shows, the following conclusions can be drawn:

1. Launching a command (similar to the exemplified task, T_2_) stops the data update during its execution and confirmation. However, if the task belonging to the data update has finished its execution before its additional time expires, the command task is executed as soon as it is received;
2. The maximum time required for performing a command occurs when the command request is launched at the beginning of the wireless transfer of the longest periodic refresh task from sequence S, needing the maximum t_1E_ time for T_1_ to perform successfully (as t_1E_ may vary from 0 to t_1Emax_ and is reached when the number of iterations has the value before timeout, t_1Smax_ is met when t_1Emax_ is met):

t _2max_ = (t_1C_ + t_1Smax_) + t_2._ (3)

This time interval is taken into consideration when deciding if it is tolerant enough while operating the structure monitored and controlled by the described system.

The system was implemented and installed at Zeicani micro hydropower plant, Hidroelectrica, Hațeg, Romania. Micro plant monitoring and control is performed from a command point situated more than 8 km away from the micro hydropower plant. The geographic area around the plant provides a sufficiently stable GSM signal, so the modem placed within the micro hydropower plant is equipped with only one conventional GSM antenna. At the command point level, a system of two GSM antennae is used, as the geographical conditions attenuate the GSM signal strength.

The Windows-compatible *MHC-01.exe* user application runs at the command point level, providing the following features:

- Real-time monitoring of the plant functioning parameters;
- Interactive display of the monitored parameters according to the MHA main diagram and the schematic diagrams of the functioning blocks;
- Command features such as generator start-up, shutdown, and increasing or decreasing the delivered power, as well as signalizing features for possible occurring faults;
- Generation of a daily archive storing the values of plant functioning parameters;
- Generation an event log entry for every start-up, shutdown, or occurring fault.

**Figure 12:**
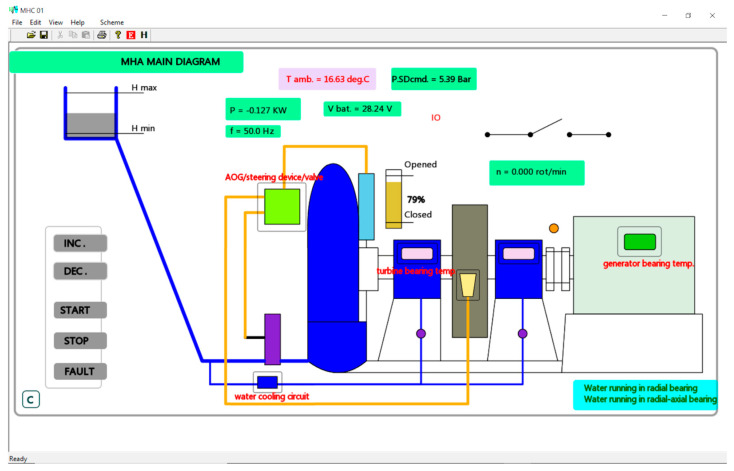
MHC-01 main window.

Figure 12 shows the main window of *MHC-01.exe*. The main monitored plant elements (turbine, generator, steering device, brake system, power network contact, etc.) and their defining state and parameters are displayed in the general diagram under generator shutdown conditions (rotational speed n = 0.0). It features a symbolic diagram corresponding to the monitored micro hydropower plant structure, also highlighting the interactions of its main functioning blocks. As pictured in the frame, critical functioning parameter values are displayed at this level (power, P; ambient temperature, T amb.; battery voltage, V bat.; rotational speed, n). The numerical value display alternates with an interactive display (including information such as oil level (open/closed) and the state of the power network contact (IO)), and significant notifications are also highlighted (regarding, for example, water running in radial and radial/axial bearings). A command panel is also featured in this window. The user can initiate generator start-up (START) or shutdown (STOP), increase delivered power (INC.), and decrease delivered power (DEC.). After initiating a command, feedback from the micro hydropower plant level is not sent until the command is executed. The user is notified of the progress of the command performance through the running messages displayed at the bottom of the main frame (“Ready”, no command has been initiated or all commands have been performed; “Command sent. Waiting <COMMAND NAME>…”, awaiting for feedback from the execution point). After receiving feedback, the interface highlights the performed command. During the functional tests, no command was initiated from the main window, and no MHA fault was been signalized.

**Figure 13:**
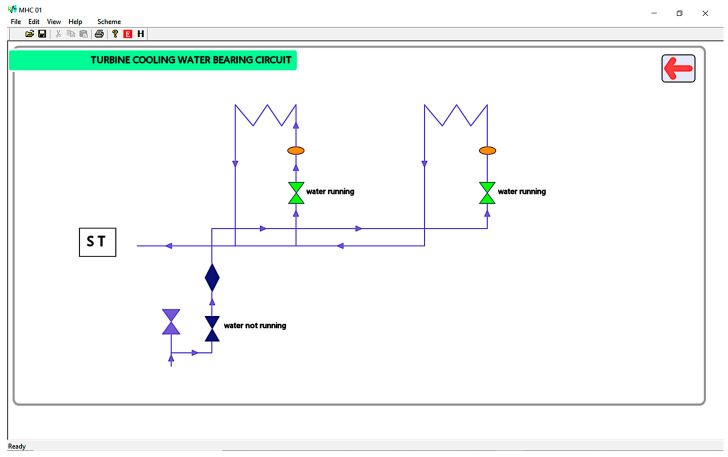
MHC-01 secondary panel for the water-cooling circuit.

A secondary panel corresponding to the water-cooling circuit is pictured in Figure 13. The panel shows a symbolic water circuit, interactively notifying the operator of water running through the diagram elements. Like all secondary panels, the window features a return button represented by a “back” arrow, which leads the user to the main frame. 

**Figure 14:**
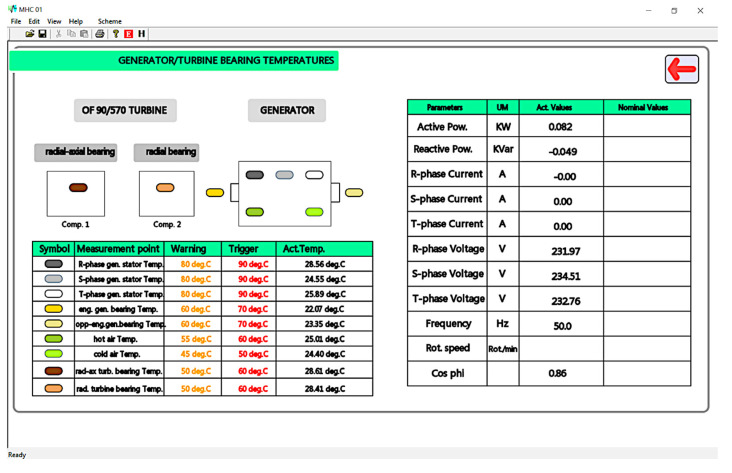
MHC-01 secondary panel for temperature measurements and main parameter values.

A list of monitored temperatures corresponding to the turbine and generator from the main diagram, together with a brief list of generally monitored parameters, can be analyzed in Figure 14. The temperature table lists the warning and fault triggering values for each entry and provides the currently measured temperature. The real-time monitored values from the general parameter list are also displayed. 

**Figure 15:**
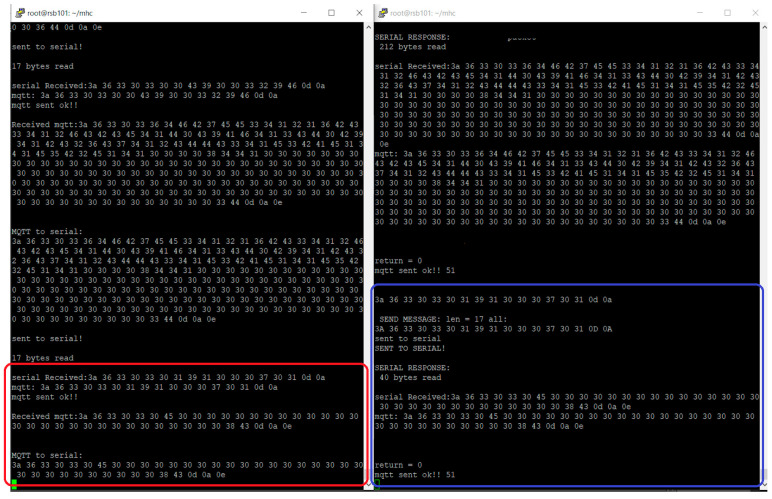
Serial and MQTT interactions between master and slave firmware.

When *MHC-01.exe* starts, data acquisition requests are serially transferred to the GSM modem (master) from the command point. These commands are received by the GSM modem located at the micro hydropower plant (slave) and serially transferred to the PC-06 data concentrator to process the command and serially transfer the response to the slave. The slave further redirects the response to the master, which further serially transfers it to the command unit to refresh the MHC-01 data. Several command and response dialogues between the GSM modems are captured through the remote connection, as shown in Figure 15, while both firmware applications are running. The sequence follows the master application serially reading a command, converting it to an MQTT message and sending it to the slave (red rectangle). Meanwhile, the slave firmware receives the command message, converts it to a serial packet, sends the packet to PC-06, reads the corresponding serial message, converts it to an MQTT message, and sends it to the master (blue rectangle). 

The MHC-01 interface update only occurs after a set of request/response message interchanges assuring that all newly acquired parameter values have been read. When choosing to view a certain panel or a certain parameter table, the values are modified in real time according to the frequency of a new data acquisition. 

**Figure A1:**
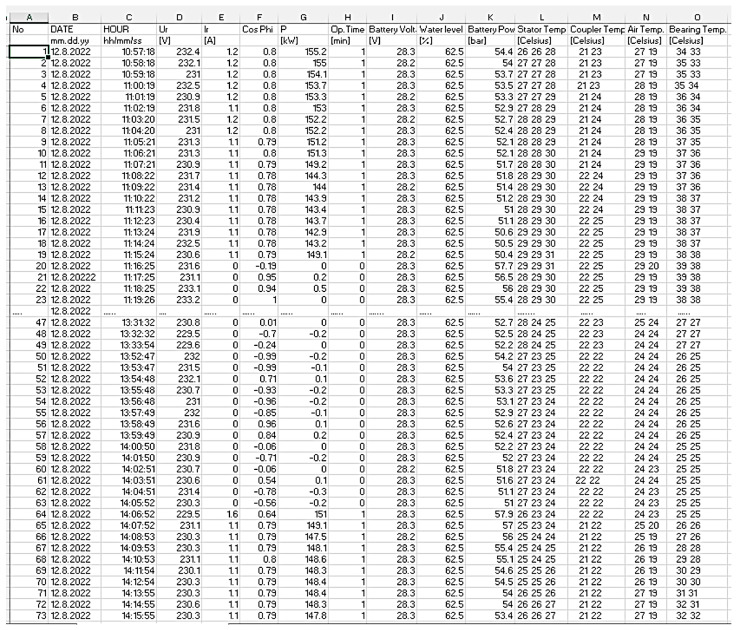
The sequences of the values from *arh_08_12_22.csv* file at 1 min time intervals were recorded during functioning tests at Zeicani micro hydropower plant, which has approximately 300 kW installed power and 0.4 kV three-phase generator output voltages. The time evolution of the various quantity values (voltages, currents, active power, power factor, temperatures, etc.) recorded in the file can be observed if the table is globally analyzed. When the power network contact (IO) is closed (Op. time = 1), active power (P) is delivered to the electric network, with a positive value larger than 0. However, if the IO contact is open (Op. time = 0), the delivered power approaches 0.

Meanwhile, while refreshing the interface, *MHC-01.exe* also updates the daily archive, a comma-separated value (csv) file available by clicking the “H” button on the main window (Figure 12). Figure A1 in Appendix A lists the values stored in the *arh_08_12_22.csv* file, which was created during the functioning tests effectuated at Zeicani micro hydropower plant.

**Figure A2:**
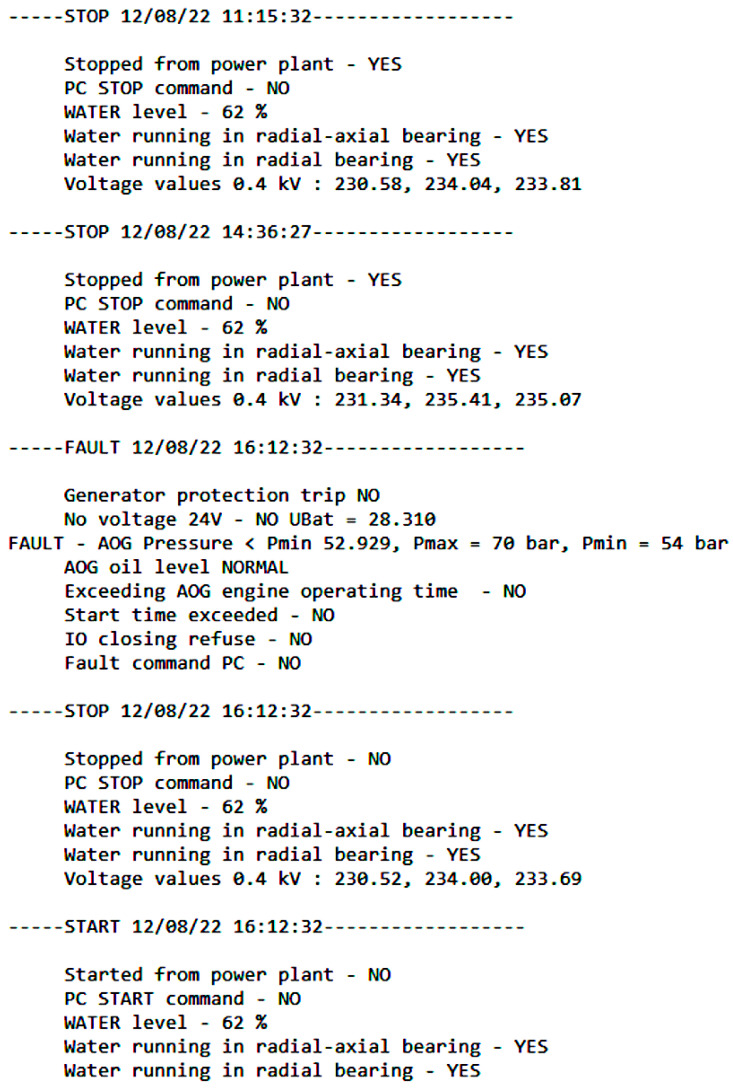
MHC-01-provided *events.log* entries from 08.12.2022. Information corresponds to the occurrence moment and the conditions triggering the event (for example, the 08.12.2022 fault was signalized due to an AOG pressure-off minimum limit), and the values of the significant parameters at the occurrence moment are mentioned for each log entry.

The events of generator start-up, shutdown, and micro hydropower plant fault signalizing are recorded in a log file that is available by pressing the “E” button on the main window of MHC-01.exe (Figure 12). The contents of the *events.log* file, which was generated while functioning tests were performed at Zeicani micro hydropower plant, are listed in Figure A2 of Appendix A. 

## 6. Conclusions

The presented application is an advantageous solution adequate for modernizing a conventionally constructed micro hydropower plant (extending its duration of use by adapting to the current requirements of exploitation), as well as for providing remote monitoring and control to micro hydropower plants without integrated wireless communication. The system is compatible with a wide range of micro hydropower plant architectures, providing customization means (for example, extending the monitored parameter lists). The diagrams used for the development of *MHC-01.exe* highlight the main functional blocks specific to micro hydropower plants in general and possible graphical adaptations that can be easily effectuated.

Furthermore, a monitoring software application can be developed to assure the control of several micro hydropower plants in a hydropower facility and, eventually, the control of several facilities in a region. At this level, a web application can be implemented on a server for easier management of several hydropower facilities, with the developed system representing a node in hierarchical hydropower control infrastructure.

Although the system and the corresponding implemented software applications are not suitable for situations requiring immediate response to commands or high-frequency data acquisition (for example, recording high-speed transient electrical events), this approach finds its utility in monitoring slow processes and initiating commands with permissive execution time.

The MHC-01 application and system can be considered a low-cost solution due to several important aspects:

- The data concentrator acquires all the information, either directly or from the monitoring devices through serial communication using several protocols, and provides it serially using the MODBUS ASCII standard protocol. Hence, the environmental sensors and transducers do not require integrated wireless communication features. Moreover, data processing is performed offline on a system that does not provide remote monitoring and control features;
- Because data gathering and processing are performed by PC-06 firmware and the MHC-01 high-level application, few data security measures can be implemented by the wireless communication infrastructure;
- Additional software resources are minimal, as the modems implement an open-source library-based application on a Linux operating system. Furthermore, no additional software is used to aid in the running of the implemented software suite;
- From a hardware point of view, the developed system architecture allows for the adaptation to the existing sensor and transducer structure within a micro hydropower plant.

The presented system can be notably improved by integrating the software infrastructure of the data concentrator in the corresponding modem software application, preserving the strategy of separating the offline coding and decoding of messages from the actual message transfer process. Online access to useful information from the system must remain restricted. Moreover, the developed structure can be installed in various configurations according to the specific situation. For example, if the controlled structure already includes a serial data concentrator, it can be directly connected to the communication modem, with data configuration customized through the software means provided by the MHC-01.exe application. 

The described architecture was developed starting from the real functioning conditions and configuration of a micro hydropower plant and is currently running at Hidroelectrica, Hațeg, Romania.

## Figures and Tables

**Table 1:** Main units of the proposed remote monitoring and control system.

Unit	Function
Protection unit (PC-05/104)	Monitors generator currents and voltages, implementing several protection functions, such as maximum current protection, overload protection, and homopolar protection. It also provides the acquired data to the data concentrator unit.
Data concentrator unit (PC-06/104)	Allows for analog value acquisition either serially or through analog inputs, in addition to assuring digital monitoring for plant digital signals of critical importance. It also collects data from all the other devices in the monitoring and control structure, including temperature adapters and rotation speed transducers. The gathered data are serially transferred by request to a command unit with the aid of the GSM modems, assuring wireless communication.
GSM structures (IFB-122)	Central processing units with an embedded GSM modem, enabling serial communication with the data concentrator and GSM communication with the other GSM terminal, which serially transfers data to the command unit. Both GSM terminals are industrial devices running in an extended temperature range of −40–85 °C.
Command unit	Any industrial device or system with a Windows-compatible operating system to allow the system to run specific software applications. It can be configured as a control unit to coordinate data updating and initiate start-up, shutdown, increase, and decrease commands. The monitored structure includes a laptop to fulfill this purpose.

## Data Availability

Data is contained within the appendices of the present paper.
